# Prevalence of *Eimeria* spp. infections and major histocompatibility complex class II *DRA* diversity in Mongolian Bactrian camels (*Camelus bactrianus*)

**DOI:** 10.3389/fvets.2023.1296335

**Published:** 2023-11-23

**Authors:** Igori Khatanbaatar, Uranbileg Nyamdolgor, Boldbaatar Chinchuluun, Khandsuren Naranbaatar, Anja Taubert, Carlos R. Hermosilla, Franz Suchentrunk, Felix Knauer, Pamela A. Burger, Gonchigoo Battsetseg

**Affiliations:** ^1^School of Veterinary Medicine, Mongolian University of Life Sciences, Ulaanbaatar, Mongolia; ^2^Institute of Veterinary Medicine, Mongolian University of Life Sciences, Ulaanbaatar, Mongolia; ^3^Institute of Parasitology, Justus Liebig University Giessen, Giessen, Germany; ^4^Research Institute of Wildlife Ecology, University of Veterinary Medicine Vienna, Vienna, Austria

**Keywords:** *Camelus bactrianus*, coccidiosis, *Eimeria cameli*, *Eimeria rajasthani*, *Eimeria dromedarii*

## Abstract

**Introduction:**

The two-humped Bactrian camel (*Camelus bactrianus*) is a large, even-toed ungulate native to the steppes of Central Asia. Domestic Bactrian camels are economically important in Mongolia and other Central Asian countries. These animals are used for transport, milk and meat production, and camel racing which is a great culture of nomads. Eimeriosis, also known as coccidiosis, is considered as an economically important parasitic diseases in Bactrian camels. There is still considerable lack of data concerning the spectrum of monoxenous *Eimeria* species, their epizootiology as well as their precise life cycles in Bactrian camels. This study was performed to determine the prevalence of *Eimeria* species in camelids from southern part of Mongolia.

**Methods:**

A total of 536 fresh camel fecal samples (*n* = 536) collected from herds located in five different Aimags (provinces) of Mongolia were examined. *Eimeria* spp. oocysts were isolated using the sugar flotation technique, and after sporulation, oocysts were identified by morphometric evaluation.

**Results:**

We identified the most common *Eimeria* species infecting Mongolian Bactrian camels: *Eimeria cameli* (22.3%), *Eimeria rajasthani* (37.3%) and *Eimeria dromedarii* (27.7%). Interestingly, mixed infections were detected in 24.8% (*n* = 133) of the samples, while 39.0% (*n* = 209) were negative for coccidian stages. To investigate the immunogenetic response of the Mongolian Bactrian camels to *Eimeria* spp. infection, we screened the genetic diversity in a functional important immune response gene of the major histocompatibility complex (MHC). We detected two polymorphic sites in the MHC class II *DRA* exon 2, which translated into one non-synonymous and one synonymous amino acid (aa) change.

**Discussion:**

The resulting aa alleles were not significantly associated with any of the three detected *Eimeria* species infections, nor could we show heterozygote advantage in non-infected Mongolian Bactrian camels. Further investigations on molecular epidemiology, *in vitro* culture, pathogenicity and host–parasite interactions will be necessary to better understand the impact of eimeriosis in Bactrian camels.

## Introduction

Extant two-humped camel species are represented by the domestic (*Camelus bactrianus*) and wild (*Camelus ferus*) species. At present, wild camels are the only wild survivors of the Camelini tribe and inhabit northwestern China and southwestern Mongolia, especially within the Outer Altai Gobi Desert (1, 2). Domestic Bactrian camels are mainly distributed in the arid desert of Asian countries, such as Mongolia, China, Russia, Kazakhstan and Iran (3). These large animals are economically important in Mongolia where they are used for transport, entertainment (camel race, camel polo), and production of derived products such as fermented milk, meat, wool and skin (4), justifying the great socioeconomic importance of camels in the country.

Eimeriosis, also known as coccidiosis, is considered an important parasitic enteric disease of camels (5), but the occurrence of monoxenous (that lives within a single host during its whole life cycle) (6) *Eimeria* species and prevalence of eimeriosis is unknown in Bactrian camels in Mongolia. Among the five species known to infect Bactrian camels, i. e. *Eimeria cameli*, *E. rajasthani*, *E. dromedarii*, *E. bactriani* and *E. pellerdyi*. Nonetheless, *E. cameli*, *E. rajasthani* and *E. dromedarii* are considered as the most pathogenic species forming first generation macromeronts as reported for other highly pathogenic *Eimeria* species of domestic ruminants and New World camelids (7–10). Several studies showed the prevalence of different *Eimeria* species in camels. As such, Chineme (11) reported a case of dromedary (*Camelus dromedarius*) coccidiosis caused by *E. cameli* in Nigeria (11). In other studies, Kawasmeh and Elbihari (12), Yagoub (13), and Kasim et al. (14) found one or more species (*E. rajasthani, E. dromedarii* and *E. cameli*) with an overall prevalence of 14% in Saudi camels (*C. dromedarius*), 17.4% in Sudanese camels (*C. dromedarius*) and 41.6% in Saudi Arabian camels (*C. dromedarius*), respectively (12–14).

The report by Tafti et al. (15) indicated that the most important and frequent pathologic lesion in the digestive tract of camels is resulting from *Eimeria* spp. infections (63% of 100 slaughtered camels) (15). These pathological findings were in close agreement with reports from Hussein et al. (16), Kasim et al. (14) and Borji et al. (17) (14, 16, 17). Several cases of eimeriosis causing enteritis and mortality rates of up to 10% in young camels have been reported in few cases (11, 18, 19). *Eimeria cameli, E. rajasthani, E. dromedarii* are pathogenic to young camel calves causing enteritis (16). Infected young animals showed wasting, debility and diarrhea without mucus or blood. Older animals shedding oocysts in their faeces did not show any serious symptoms of eimeriosis (20). Considering the fact that all *Eimeria* infections are highly host and host cell-specific and that stage-specific innate as well as adaptive immune reactions are a common feature (21, 22), it appears essential that basic research is performed on different developmental stages in the respective hosts (8, 23).

Pathogen-mediated selection has been described as a driver for genetic diversity in host immune response genes, especially in the major histocompatibility complex (MHC). The MHC class I and class II genes are responsible for encoding molecules on the cell surface that recognize and present antigens (24). Therefore, these molecules are under strong selective pressure and have an important role for the adaptive immune response and for host-pathogen co-evolution (25). Studies in Inner Mongolian Brandt’s voles showed that MHC class II diversity is maintained by rare allele advantages and fluctuating selection, and that the association between intestinal parasite load and specific MHC class II *DRB* alleles varied between geographical regions (26).

In the three extant Camelini species (*C. bactrianus*, *C. ferus*, *C. dromedarius*), the MHC is located on chromosome 20, with the class II region located closer to the centromere and the class I more distant (27). In camels, unexpectedly low diversity has been described in the MHC class II loci (27) as well as in all functional different groups (adaptive and innate) of immune response genes (28). So far, no study about the immunogenetic response in camels to intestinal parasite infection has been conducted.

To fill this knowledge gap, the aim of this study was to determine the prevalence of *Eimeria* species in domestic Bactrian camels in southern Mongolia as well as their adaptive immunogenetic response to *Eimeria* spp. infections. Due to its functional importance, specifically in connection with parasite infection, we focused on the exon 2 coding sequence of the MHCII *DRA* locus and investigated pathogen-mediated immunogenetic diversity in Mongolian Bactrian camels infected or non-infected with *Eimeria* spp.

## Materials and methods

### Sample collection and study field area

**Figure 1:**
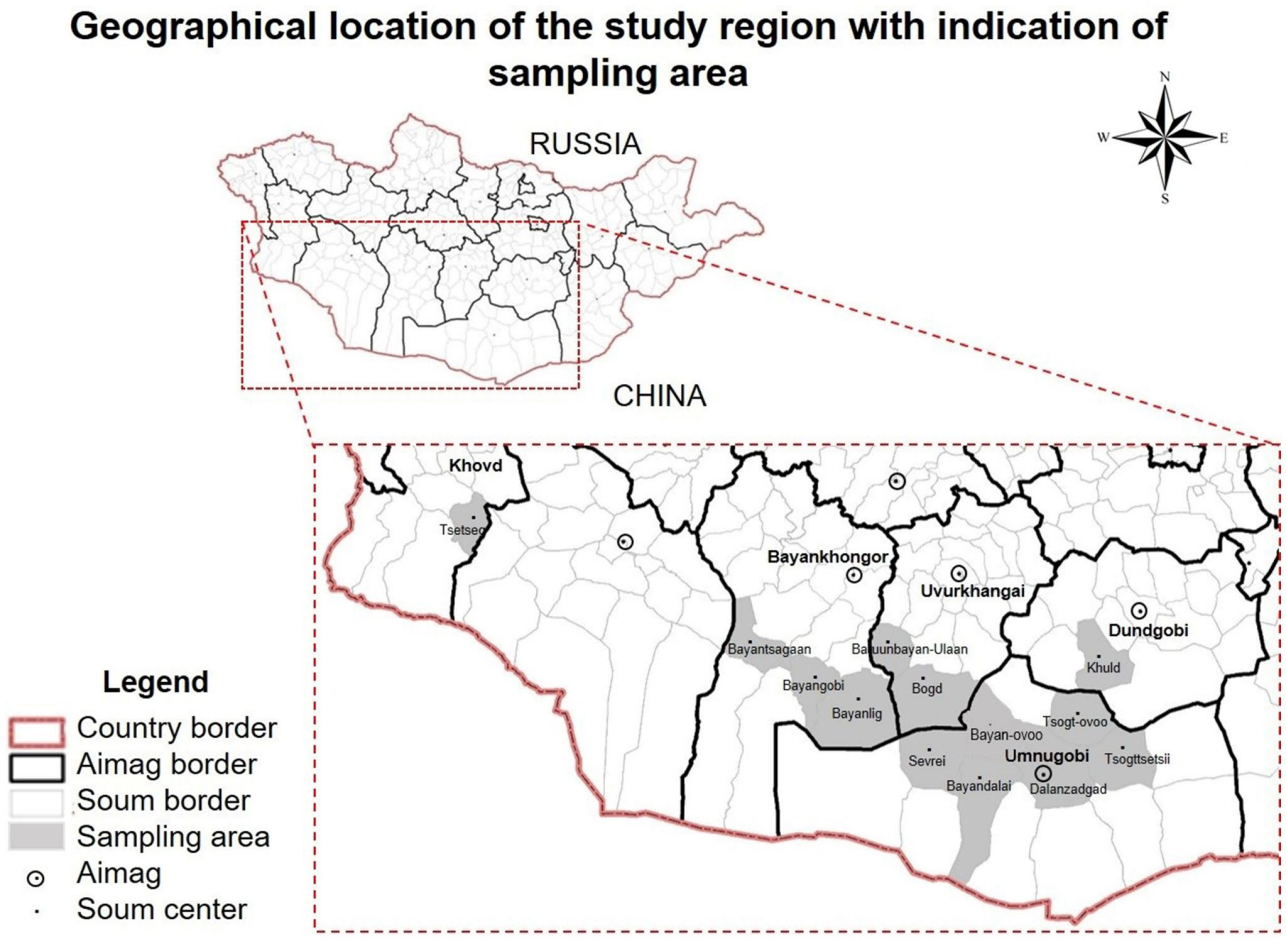
Geographical location of the study region with indication of Bactrian camel (*Camelus bactrianus*) sampling areas. Khovd, Bayankhongor, Uvurkhangai, Dundgobi, Umnugobi Aimags of Mongolia. This map has been provided by the ArcGIS 10.2 program.

Fecal samples were collected from 536 Mongolian Bactrian camels in the Umnugobi-, Bayankhongor-, Uvurkhangai-, Dundgobi– and Khovd Aimags, chosen at random (Figure 1). Collected samples were put separately into closed plastic containers and identified with numbers. In addition, 100 EDTA peripheral blood samples were collected in parallel to the fecal sample collection. All experimental protocols were approved by the Animal Care and Use Committee, Institute of Veterinary Medicine, Mongolian University of Life Sciences (MULS) (Agreement Number № MEBUS-16/01/05). Samples from local provinces were taken from live animals with official permission and under the supervision of the Provincial Veterinary Organization in accordance with the regulation of the Animal ethic committee, MULS.

The examined Bactrian camels followed traditional husbandry practices, with animals grazing during daytime. Camels were mainly crossbreeding and indigenous. For representative reasons, geographical locations of camels sampled are shown in Figure 1.

### Detection and identification of *Eimeria* oocysts

All fecal samples were examined by sugar flotation technique for isolation of camelid *Eimeria* spp. oocysts. Briefly, 3 g of fecal material were weighted and placed into a beaker and 15 mL of saturated sucrose solution (Sheather’s solution, specific gravity = 1.28) were added and homogenized. Thereafter, the fecal suspension was transferred into a 15 mL centrifuge tube and centrifuged at 2,000 rpm for 10 min at room temperature (RT) (29–37).

Samples were investigated by means of light microscopy and all oocysts within the microscope slides were considered in this study.

For species identification, oocysts from each individual sample were allowed to sporulate in 2.5% potassium dichromate under constant oxygenation (38). *Eimeria* species identification was based on the morphological and morphometric features of sporulated oocysts such as the size, shape, colour and texture of oocyst wall, presence or absence of micropyle, polar cap, among others, with the aid of taxonomic keys according to Levine and Ivens (1970), (6, 29, 39).

### Analysis of the major histocompatibility complex (MHC) class II *DRA* exon 2

DNA was extracted using the QIAmp® blood mini kit (Qiagen, Vienna, Austria) from 100 EDTA peripheral blood samples following the manufacture’s instruction. The 246 bp long MHCII *DRA* exon 2 was amplified with the camel specific DNA primer pairs (DRA-ex2-F-TGAGAATTTTGGGTTTGCTTATGGCA/ DRA-ex2-R-CCTCTGAGCAACACG AACGTC CTTCA) with an annealing temperature of 57°C (27). The PCR reactions were performed in a reaction volume of 15 μL including 0.2 mM dNTPs, 25 mM MgCl_2_, 0.5 μM of forward and reverse primer, 1x Amplitaq Gold buffer, and 0.5 U of Amplitaq Gold Hotstart polymerase (ThermoFisher Scientific, Vienna, Austria). Please note that we tried to sequence also MHCII *DRB* exon 2 following Plasil et al. (27), however, only few samples yielded PCR products, which might be due to the longer amplicon of 852 bp (27). Successfully amplified PCR products were purified with FastAP™ and Exonuclease I (ThermoFischer Scientific) following the manufacture’s guide for PCR product clean-up prior to sequencing. The purified PCR products were Sanger sequenced in both directions with the BigDye™ Terminator v3.1 Cycle Sequencing Kit (ThermoFischer Scientific) on an ABI sequencer at the Research Institute of Wildlife Ecology, Vetmeduni Vienna, Austria. Sequences were visualised, aligned and translated into amino acids (aa) using CodonCode Aligner 11.0.1 (CodonCode Cooperation, Centerville, USA). We applied DNAsp 5.10.1 (40) to phase ambiguous (heterozygous) sequences and to determine haplotype (Hd) and nucleotide diversity (Pi).

To test for potential associations between *Eimeria* infection and the MHCII *DRA* exon2 aa alleles as well as for heterozygote advantage, we applied the generalized linear model with a logit link function in IBM® SPSS® Statistics version 29.0.1. (IBM Corp., Armonk, NY, USA). We tested six models with each of the three detected *Eimeria* species as binary dependent variable and the aa alleles, heterozygosity, gender and the respective other *Eimeria* spp. (Table 1) as binary, location (soum) as categorical, and age as continuous predictor variables, respectively. We applied strict Bonferroni correction for multiple model (k = 6) testing for a nominal alpha error of 0.05. In case of a significant effects of co-infection with two *Eimeria* species (*p* < 0.0083, Bonferroni corrected) we used Crosstabs statistics in SPSS to calculate the association coefficient Phi, which is a chi-square-based measure of association between nominal data (41).

**Table 1:** Sample information for genotyped (MHC II *DRA* exon 2) individuals.

ID	Genotype MHCII *DRA* exon 2 pos.58/143	aa* allele	*E. cameli*	*E. rajasthani*	*E. dromedarii*	soum	gender	age
Т-1	TT/GG	0	0	0	0	Bayan-Ovoo	female	5
Т-2	TA/GT	2	0	0	0	Bayan-Ovoo	female	2
Т-3	TT/TT	0	0	0	0	Bayan-Ovoo	female	6
Т-5	TT/GG	0	0	0	0	Bayan-Ovoo	female	6
Т-6	TT/GG	0	0	0	0	Bayan-Ovoo	female	10
Т-7	AA/TT	1	0	0	0	Bayan-Ovoo	female	8
Т-8	AA/TT	1	0	0	0	Bayan-Ovoo	female	9
Т-9	TT/GG	0	0	0	0	Bayan-Ovoo	female	10
Т-10	TA/GG	2	0	0	0	Bayan-Ovoo	female	1
Т-11	TA/TT	2	0	0	0	Bayan-Ovoo	female	6
Т-12	TT/GG	0	0	0	1	Bayan-Ovoo	female	6
Т-13	TT/GG	0	0	1	1	Bayan-Ovoo	female	9
Т-14	TT/GG	0	1	0	1	Bayan-Ovoo	female	7
Т-15	TT/GG	0	1	1	0	Bayan-Ovoo	female	7
Т-23	TA/TT	2	1	1	0	Bayan-Ovoo	female	1
Т-24	TA/GT	2	0	0	0	Bayan-Ovoo	male	1
Т-25	TA/GT	2	0	0	0	Bayan-Ovoo	female	1
Т-26	TA/GT	2	0	0	0	Bayan-Ovoo	female	1
Т-27	TA/GT	0	0	0	0	Bayan-Ovoo	male	1
Т-28	TA/GT	2	0	0	0	Bayan-Ovoo	male	1
Т-29	TA/GT	2	0	0	0	Bayan-Ovoo	female	1
Т-30	TA/GT	2	0	0	0	Bayan-Ovoo	female	1
Т-31	AA/TT	1	0	0	0	Tsogttsutsii	female	10
Т-32	TA/GT	2	0	1	1	Tsogttsutsii	male	1
Т-33	TA/GT	2	0	1	1	Tsogttsutsii	female	8
Т-34	TA/GT	2	1	0	1	Tsogttsutsii	female	8
Т-35	AA/TT	1	0	0	1	Tsogttsutsii	female	8
Т-36	AA/TT	1	0	0	1	Tsogttsutsii	female	6
Т-37	TT/GG	0	0	0	1	Tsogttsutsii	female	6
Т-38	TA/GT	2	0	0	1	Tsogttsutsii	female	7
Т-46	AA/TT	1	0	0	0	Tsogttsutsii	male	1
Т-54	TT/GG	0	0	0	0	Tsogttsutsii	female	14
Т-55	TA/GT	2	0	0	0	Tsogttsutsii	female	15
Т-56	TA/TT	2	0	0	0	Tsogttsutsii	female	14
Т-57	TA/GT	2	0	0	0	Tsogttsutsii	female	6
Т-58	TT/GG	0	0	0	0	Tsogttsutsii	female	14
Т-59	TT/TT	0	0	0	0	Tsogttsutsii	female	20
Т-60	TA/TT	2	0	0	0	Tsogttsutsii	female	14
Т-61	TA/GT	2	0	0	0	Tsogttsutsii	female	6
Т-62	AA/TT	1	0	0	0	Tsogttsutsii	female	14
Т-63	TA/GT	2	0	0	0	Tsogttsutsii	female	6
Т-64	TA/GT	2	0	0	0	Dalanzadgad	male	1
Т-65	TT/GT	0	0	0	0	Dalanzadgad	female	1
Т-66	TA/GT	2	0	0	0	Dalanzadgad	female	1
Т-67	TA/GT	2	0	0	0	Dalanzadgad	female	1
Т-68	TT/GG	0	1	1	0	Dalanzadgad	male	1
Т-69	TA/TT	2	0	0	0	Dalanzadgad	male	1
Т-70	TT/GT	0	0	0	0	Dalanzadgad	female	1
Т-71	TA/GT	2	0	0	1	Dalanzadgad	male	1
Т-72	TT/GT	0	0	0	1	Dalanzadgad	female	1
Т-73	TA/GT	2	1	1	0	Dalanzadgad	female	1
Т-74	TA/TT	2	0	0	1	Dalanzadgad	female	12
Т-76	AA/TT	1	0	0	1	Dalanzadgad	female	5
Т-77	AA/TT	1	0	0	1	Dalanzadgad	female	10
Т-78	AA/TT	1	0	0	0	Dalanzadgad	female	12
Т-79	TA/TT	2	0	0	0	Dalanzadgad	female	12
Т-80	TA/GT	2	0	0	0	Dalanzadgad	female	5
Т-81	TA/TT	2	0	0	0	Dalanzadgad	female	5
Т-82	TA/TT	2	0	0	0	Dalanzadgad	female	5
Т-84	TA/GT	2	0	0	0	Dalanzadgad	female	14
Т-85	TA/GT	2	0	0	0	Dalanzadgad	female	6
Т-86	TA/TT	1	0	0	0	Dalanzadgad	female	5
Т-87	TA/GT	2	0	0	0	Dalanzadgad	female	10
Т-88	TA/TT	2	0	0	0	Dalanzadgad	female	21
Т-89	TT/TT	0	0	0	0	Dalanzadgad	female	7
Т-90	TA/GT	2	0	0	0	Dalanzadgad	female	6
T177	TA/TT	2	0	1	0	Bayangobi	female	3
T178	TT/GT	2	0	0	0	Bayangobi	female	11
T179	TT/GT	2	0	0	0	Bayangobi	female	1
T180	TT/GT	2	0	0	0	Bayangobi	female	9
*Eimeria* spp. prevalence (%)			6 (8.6)	8 (11.4)	15 (21.4)			

*0: homozygote H1 (FF); 1: homozygote H2 (YY); 2: heterozygote H1/H2 (FY).

## Results

### *Eimeria* spp. infection in Mongolian Bactrian camels

**Figure 2:**
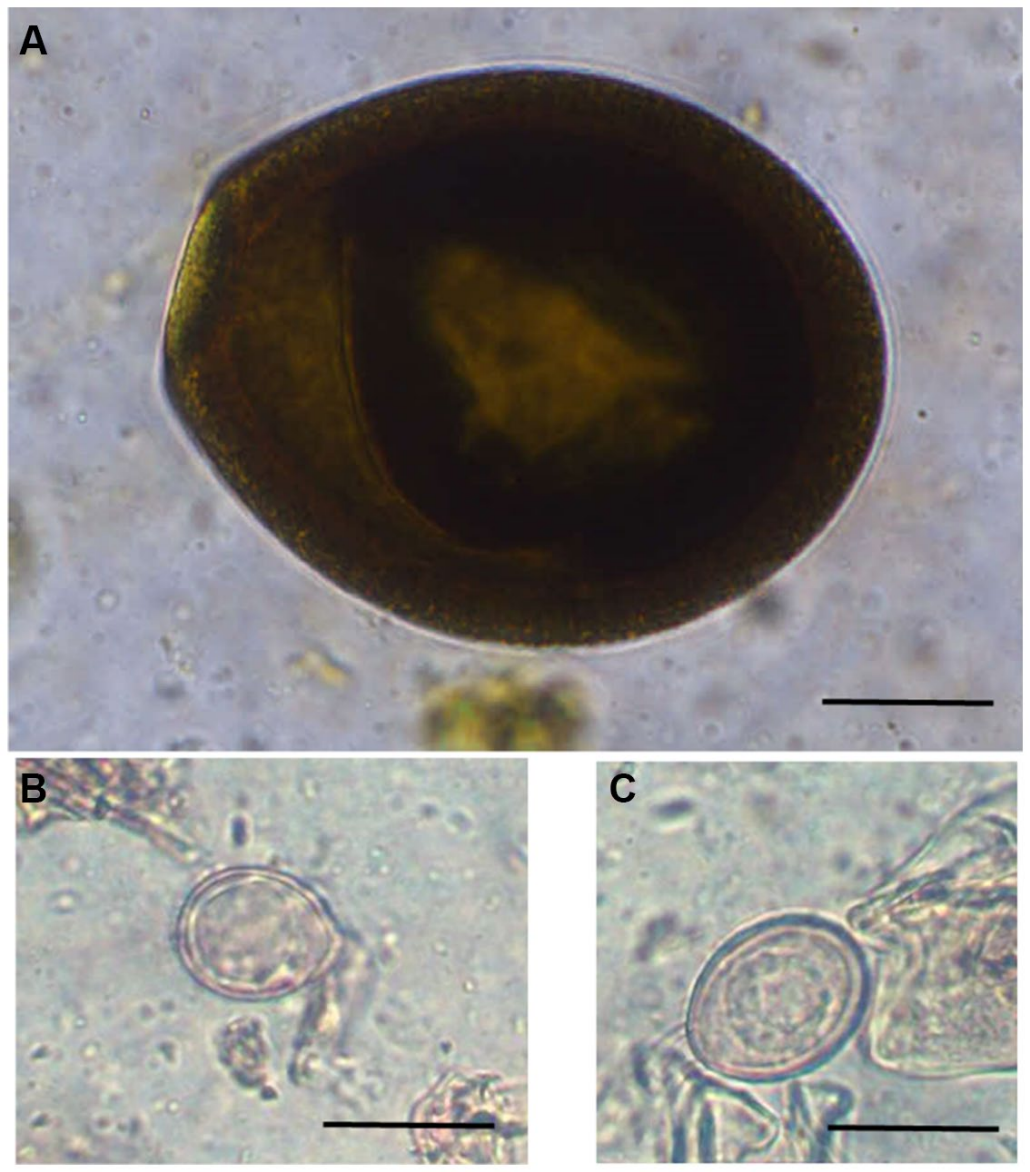
Photomicrographs of different unsporulated *Eimeria* oocysts of Bactrian camels (*Camelus bactrianus*) in Mongolia. **(A)** *Eimeria cameli*. The oocysts were truncated ovoid, dark brown to black in colour. The oocyst wall was composed of 3 layers: outer, dark brown in colour with tiny projections. The middle layer was thin smooth and yellowish in colour. The inner layer was dark brown. Length x width 96.5 × 82 μm, **(B)** *Eimeria dromedarii*. The oocyst shape ranged from subspherical to ovoid, with rough walls composed of two distinct layers: outer, pale yellow and inner, dark green. Length x width = 23 × 17 μm and, **(C)** *Eimeria rajasthani*. The oocysts were ellipsoidal in shape with smooth walls that were composed of two layers: outer, pale yellow and inner, yellowish green in colour. The size length x width 29 × 21.6 μm. Scale bar = 25 μm.

In total, 327 fecal samples (*n* = 327) had *Eimeria* oocysts with an overall prevalence of 61% in Bactrian camels from Bayankhongor-, Uvurkhangai-, Umnugobi-, Dundgobi– and Khovd Aimags. *Eimeria* parasites were found in all five investigated Aimags (Figure 1). Three different camelid-specific *Eimeria* species were identified, being *E. rajasthani* 200 (37.3%) and *E. dromedarii* 149 (27.7%) the most prevalent ones, followed by *E. cameli* 120 (22.3%). In 209 samples (39%) no *Eimeria* oocysts were observed (Table 2; 3). Mixed *Eimeria* spp. infections with two or three *Eimeria* species, were detected in 133 samples (24.8%) (Table 3). Out of 327 positive samples, 194 (36.1%) samples presented single infection, 125 (23.3%) samples had two species, and only 9 (1.6%) samples had mixed infections with all three species (see Figure 2).

**Table 2:** Occurrence of *Eimeria* spp. in Mongolian Bactrian camels.

Name of Aimag	Soum	Sample number	*E. cameli*		*E. rajasthani*		*E. dromedarii*		Mixed infection	
			Positive	%	Positive	%	Positive	%	Positive	%
Bayankhongor	Bayantsagaan	82	23	28.05	29	35.3	15	18.29	16	19.51
	Bayanlig	60	10	16.67	45	75	30	50	31	51.6
	Bayangobi	14	4	28.57	7	50	9	64.29	8	57.14
**Total**		156	37	23.72	81	51.92	54	34.62	55	35.9
Uvurkhangai	BaruuBayan-Ulaan	85	20	23.53	33	38.82	26	30.59	21	24.71
	Bogd	17	10	58.82	6	35.29	1	5.88	3	17.65
**Total**		102	30	29.41	39	38.24	27	26.47	24	23.53
Umnugobi	Sevrei	65	23	35.38	26	40	23	35.38	18	27.69
	Dalanzadgad	37	6	16.21	6	16.21	12	32.43	6	16.21
	Tsogttsutsii	49	3	6.12	8	16.32	17	34.69	9	18.36
	Tsogt-Ovoo	21	1	4.76	1	4.76	2	9.52	1	4.76
	Bayandalai	32	5	15.63	17	53.13	1	3.13	4	12.5
	Bayan-Ovoo	30	4	13.33	7	23.33	9	30	8	26.66
**Total**		234	42	17.94	65	27.77	64	27.35	46	19.65
Dundgobi	Khuld	19	10	52.63	4	21.05	1	5.26	4	21.05
Khovd	Tsetseg	25	1	4	11	44	3	12	4	16
**Grand total**		536	120	22.3	200	37.3	149	27.7	133	24.8

**Table 3:** Mixed *Eimeria* spp. infection in Mongolian Bactrian camels.

Name of Aimag	Soum	total	Mixed infection									
			Number of mixed infections		non infected		single species		two species		three species	
			Pos.	%	Neg.	%	Pos.	%	Pos.	%	Pos.	%
Bayankhongor	Bayantsagaan	82	16	19.5	33	40.2	33	40.2	14	17	2	2.4
	Bayanlig	60	31	51.6	7	11.6	22	36.6	29	48.3	2	3.3
	Bayangobi	14	8	57.1	2	14.3	4	28.6	8	57.1		
Total		156	55	35.2	42	26.9	59	37.8	51	32.6	4	2.6
Uvurkhangai	Baruunbayan-Ulaan	85	21	24.7	31	36.4	33	38.8	18	21.1	3	3.52
	Bogd	17	3	17.6	3	17.6	11	64.7	3	17.6		
Total		102	24	23.5	34	33.3	44	43.1	21	20.5	3	2.9
Umnugobi	Sevrei	65	18	27.6	11	16.9	36	55.3	18	27.6		
	Dalanzadgad	37	6	16.21	21	56.8	10	27.02	5	13.51	2	5.4
	Tsogttsutsii	49	9	18.3	30	61.2	10	20.4	9	18.3		
	Tsogt-Ovoo	21	1	4.76	18	85.7	2	9.5	1	4.7		
	Bayandalai	32	4	12.5	13	40.6	15	46.8	4	12.5		
	Bayan-Ovoo	30	8	26.6	18	60	4	13.3	8	26.6		
Total		234	46	19.7	111	47.4	77	32.9	45	19.2	2	0.9
Dundgobi	Khuld	19	4	21	8	42.1	7	36.8	4	21		
Khovd	Tsetseg	25	4	16	14	56	7	28	4	16		
Grand total		536	133	24.8	209	39.0	194	36.2	125	23.3	9	1.7

### MHC II *DRA* diversity in Mongolian Bactrian camels with *Eimeria* spp. infections

**Figure 3:**
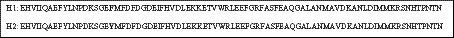
MHCII *DRA* exon 2 amino acid haplotypes identified in Mongolian Bactrian camels. The polymorphic amino acid change is highlighted in bold.

We successfully amplified and sequenced the 246 bp long MHC class II *DRA* exon 2 in 70 (out of 100) samples (Table 1). We screened the *DRA* exon 2 for genetic diversity and detected two polymorphic nucleotides (nt) at the positions nt = 58 and nt =143 in the 246 bp long fragment. Phasing the 70 individual sequences resulted in three haplotypes (h = 3) with a haplotype (gene) diversity Hd = 0.620 (± 0.018), nucleotide diversity Pi = 0.004 (± 0.00006) and an average number of nucleotide differences k = 0.983. At position nt = 58, the change from T to A (T58A) led to a non-synonymous amino acid (aa) change from phenylalanine (F) to tyrosine (Y), both hydrophobic, while the nucleotide change G143T was synonymous (no aa change). This resulted in two different aa alleles (haplotypes; H1 and H2) identified in Mongolian Bactrian camels as shown in Figure 3. While 19 camels were homozygous for H1 and 11 individuals for H2, respectively, the majority of 40 Bactrian camels was heterozygous and harboured both aa alleles of the MHCII *DRA* exon 2. The complete sample, genotype and aa allele information is presented in Table 3. The sequence alignment of MHCII *DRA* exon 2 for all Bactrian camel samples is provided in Supplementary file S1.

We investigated a potential statistical effect between the MHC *DRA* exon 2 aa alleles H1, H2 or H1/H2 on the three different *Eimeria* spp. infections in the Bactrian camels, using a generalised linear model approach. However, we could not identify any significant (*p* < 0.0083 after Bonferroni correction) association between the MHCII *DRA* aa alleles and any of the *Eimeria* spp. infections (Table 4). Similarly, we did not detect a significant effect of the homozygote or heterozygote genotypes on *Eimeria* spp. infections, respectively. However, we identified a significant (*p* = 0.003) positive effect with a moderate association coefficient Phi = 0.532 (*p* < 0.001) between two *Eimeria* species, *E. cameli* and *E. rajasthani*, independent from all other tested predictor variables and factors, i. e., location, age or gender did not show any significant effect on the prevalence of *E. cameli* and *E. rajasthani* in the respective models under strict Bonferroni correction (Table 4).

**Table 4:** Parameter estimates of the generalized linear model testing between MHCII *DRA* exon 2 amino acid (aa) alleles and *Eimeria* spp. infections.

			Dependent variable								
			*E. cameli*			*E. rajasthani*			*E. dromedarii*		
	Parameter estimates		B	Std.err	Sig.	B	Std.err	Sig.	B	Std.err	Sig.
Predictor variables		Intercept	0.497	1.705	0.771	−3.681	1.938	0.058	−0.225	1.334	0.866
	Eimeria sp.	*E. cameli*	_	_	_	3.473	1.166	0.003	0.047	1.214	0.969
		*E. rajasthani*	3.467	1.166	0.003	_	_	_	1.301	1.115	0.243
		*E. dromedarii*	0.440	1.435	0.759	1.141	1.281	0.373	_	_	_
	aa allele	H0	−0.847	1.332	0.525	−0.414	1.218	0.734	−0.953	0.764	0.213
		H1	18.726	23,468	0.999	19.736	21,802	0.999	−1.233	0.849	0.147
		H2	0^a^	.	.	0^a^	.	.	0^a^	.	.
		homozygous	−0.586	1.342	0.662	0.173	1.112	0.877	1.068	0.664	0.108
		heterozyous	0^a^	.	.	0^a^	.	.	0^a^	.	.
	Soum	Bayan-Ovoo	−0.472	2.022	0.815	0.874	1.658	0.598	1.850	0.93	0.047
		Bayangobi	20.818	32,680	0.999	−1.598	1.906	0.402	22.21	37,402	1.000
		Dalanzadgad	−0.350	2.165	0.817	1.529	1.786	0.392	1.093	0.821	0.183
		Tsogttsutsii	0^a^	.	.	0^a^		.	0^a^	.	.
	Sex	female	−1.325	1.803	0.463	0.771	1.345	0.566	−0.745	1.144	0.515
		male	0^a^	.	.	0^a^		.	0^a^	.	.
		age	0.141	0.201	0.485	0.191	0.184	0.298	0.086	0.086	0.321

B: unstandardised coefficient (slope of the line between the predictor and dependent variables).

a set to zero by SPPS because this parameter is redundant (can be explained by the other variables).

Sig.: *p*-values; highlighted in bold if significant (*p* < 0.0083) after strict Bonferroni correction for multiple model (k = 6) testing with a nominal alpha error of 0.05.

## Discussion

Eimeriosis, in Camelini worldwide has been recently summarized (5). However, no reports from Mongolia were included since there were no available studies at the time. Here, we report the prevalence of *Eimeria* spp. infections in southern Mongolia. Due to the rather high prevalence of camel coccidiosis, it could be assumed that *Eimeria* infections are widely spread in the country, and it may play an important role as underestimated subclinical or clinical disease affecting the growth rate performance of mainly young Mongolian Bactrian camels as reported for other hosts. *Eimeria* spp. prevalence up to 50% was reported in Bactrian camels from Inner Mongolia in China (42). In total, three monoxenous *Eimeria* species were found: *E. dromerdarii*, *E. rajasthani* and *E. cameli*, which are considered pathogenic for camels (20). Mixed infections with two or three species were here observed, presenting a higher frequency then previously reported by Yakhchali and Athari (2010) (43). The authors described the identification of four *Eimeria* species including *E. bactriani* (52.4%), *E. cameli* (19.3%), *E. pellerdyi* (15.6%) and *E. dromedarii* (12.5%) and mixed infections (up to four *Eimeria* species) in 10.54% of investigated camels.

Regarding pathogeny of camel coccidiosis several studies confirm its importance. Iyer et al. (44) reported an outbreak of gastroenteritis in camels in Punjab, India, affecting hundreds of camels with 1–40% mortality during summer months (44). Two dead camels were examined at necropsy. Gastroenteritis was the predominant finding and affected abomasum, duodenum, and cecum; jejunum and ileum were not examined. Endogenous stages (schizonts, gamonts, and oocysts) were detected in duodenum, and cecum (15, 45, 46). Same conclusion applies to a similar case of haemonchosis and *E. cameli*-associated gastroenteritis in one year old camel from India (45). Rangarao and Sharma (47) noted diarrhea-associated with the presence of *E. rajasthani* oocysts in all eight calves in India (47). An eimeriosis-like illness was diagnosed histologically in 27 of 38 camels submitted in 1996 for *postmortem* examination to the Central Veterinary Research Laboratory (CVRL), Dubai, UAE (48). Of these 27, illness was severe in 21 and mild in 6 animals, respectively. Severe hemorrhagic enteritis with eosinophilia of small intestine (mostly jejunum and ileum and rarely duodenum) was associated with numerous stages of *E. cameli* whereas large intestines were not affected.

Camel eimeriosis has mostly been associated with younger camels (20, 49). It is an important disease in pre-weaned and recently weaned camels (20). While nearly animals of all ages are exposed to infectious sporulated *Eimeria* oocysts in the environment, they may not show obvious signs of the disease. In the majority of the hosts, the parasite coexists causing minimal damage to the infected host (20). Clinical eimeriosis usually occurs if the host is subject to a heavy infection, with high number of infectious oocysts ingested, or if its resistance is lowered (20), and its immune status is not adequate to cope with a coccidian infection.

Another critical time for the infection of camels could be the time immediately preceding the period of dryness (peak from July to October), when the short winter rains cause camels to crowd from the surrounding desert to limited water holes and springs in oases (12), like in Saudi Arabia, potentially promoting the spread of the disease. Further studies with camel *Eimeria* spp., including molecular characterization and establishment of suitable *in vitro* culture systems will allow detail investigations on sporozoite*-*host cell interactions and early host innate immune reactions as reported for ruminant eimeriosis (8, 38).

Concerning immunogenic response, in the 246 bp exon 2 of the MHCII *DRA* locus sequenced in 70 Mongolian Bactrian camels infected or non-infected with *Eimeria* spp. we detected the same two nucleotide polymorphisms as described before in a global set of Bactrian camels (27). These synonymous and non-synonymous polymorphisms, which produce three different aa alleles are also shared between dromedaries (*C. dromedarius*) and wild camels (*C. ferus*) (27). The frequency (0.57) of the heterozygous allele in the here investigated Mongolian Bactrian camels was similar to the frequency described in global Bactrian camels (0.53), lower than in wild camels (0.63) and higher than in dromedaries (0.32) (27). MHC diversity is often maintained by pathogen-mediated balancing selection. It is generally assumed that heterozygous individuals have an advantage, e.g., higher fitness than individuals that are homozygous at the same locus (50, 51). Although we observed twice as many heterozygous individuals (*n* = 40) for the aa alleles at position nt 58 than homozygotes for either the reference (*n* = 19) or alternative (*n* = 11) allele, we could not identify a statistically significant heterozygosity effect on the *Eimeria* spp. infection, in terms of prevalence. No evidence for MHC class II *DRB* heterozygote advantage in relation to intestinal parasite infection has been described in Brandt’s voles from Inner Mongolia (26). The lack of such a heterozygosity effect in our study might also be explained by the relatively low number (*n* = 70) of successfully phenotyped (*Eimeria* spp. infection) and genotyped (MHCII *DRA* exon 2) samples. In addition, the *Eimeria* infection status was evaluated in a qualitative way, i.e., presence or absence, while a quantitative assessment (i.e., intensities of infection) would have provided more refined infection data to be included into our statistical models. Contrary to many other species, Old World camels and specifically Bactrian camels have a low number of MHC II *DRB* alleles. In fact, the *DRB* exon 2 showed only four alleles in 43 previously investigated Bactrian camels, which translated into two haplotypes at the amino acid level with one haplotype present in 74% of the samples (27). As our attempt to amplify MHCII *DRB* exon 2 unfortunately failed, we cannot exclude that we might have identified new *DRB* alleles in the investigated Mongolian Bactrian camels. However, considering our findings of the *DRA* exon 2 identifying exactly the previously described alleles (27), we probably might neither expect novel *DRB* alleles in the population. We did not find any age-associated effect on *Eimeria* spp. prevalence, even though older individuals may be expected to have higher prevalence than younger ones, simply due to the longer lifetime that accumulates their change of infection. This might suggest that adult may cope with the infection successfully and clear it, possibly also in connection with their overall immunogenetic status.

Interestingly, we detected a moderate positive association between the two *Eimeria* species *E. cameli* and *E. rajasthani*. Co-evolution between two parasites (*E. cameli* and *E. rajasthani*) has been observed, and these two species were dominant in Mongolian camels. While at a low dose inoculate a linear reproduction is observed, at higher doses the reproduction of the parasite becomes impaired (the so-called ‘crowding effect’), mainly due to the damage of cells or lower availability of nutrients (52). Resistance of the host to a pathogen is a very important factor in the evolutionary arms race between host and parasite. Although the host evolves at a much slower rate than the parasite is capable of, the host has developed ways to reduce susceptibility to infection. We argue that studying questions of host–parasite interaction in camelids can be well approached with an *Eimeria* parasite infection system in Bactrian camels.

In conclusion, this study revealed that *E. cameli, E. rajasthani,* and *E. dromedarii* infections frequently occur in Mongolian Bactrian camel. Given that clinical and subclinical *Eimeria* spp. infections are well known to dampen camel production, regular monitoring including diagnosis of species, quantitative description of the parasite infection load, and MHC and other immunogenetic loci could help to prevent future *Eimeria*-induced economic losses in Mongolian camel rearing.

## Data availability statement

The original contributions presented in the study are included in the article/Supplementary material, further inquiries can be directed to the corresponding author.

## Ethics statement

The animal studies were approved by Animal Care and Use Committee, Institute of Veterinary Medicine, Mongolian University of Life Sciences (Agreement Number № MEBUS-16/01/05). The studies were conducted in accordance with the local legislation and institutional requirements. Written informed consent was obtained from the owners for the participation of their animals in this study.

## Author contributions

IK: Conceptualization, Formal analysis, Funding acquisition, Writing – original draft, Data curation, Investigation. UN: Data curation, Investigation, Visualization, Writing – review & editing. BC: Data curation, Investigation, Writing – review & editing. KN: Data curation, Formal analysis, Investigation, Writing – review & editing. AT: Conceptualization, Supervision, Validation, Writing – review & editing. CH: Conceptualization, Supervision, Validation, Writing – review & editing. FS: Formal analysis, Methodology, Writing – review & editing. FK: Methodology, Software, Validation, Writing – review & editing. PB: Conceptualization, Formal analysis, Funding acquisition, Project administration, Resources, Supervision, Writing – original draft. GB: Conceptualization, Funding acquisition, Supervision, Writing – review & editing.
