# Supplementary material for: Prevalence of Eimeria spp. infections and major histocompatibility complex class II DRA diversity in Mongolian Bactrian camels (Camelus bactrianus)
Source: Front Vet Sci. 2023 Nov 23;10:1296335. doi: 10.3389/fvets.2023.1296335 (PMC10701389; doi:10.3389/fvets.2023.1296335)
Supplement: Supplementary file 1 [file Table_1.DOCX]

Supplementary Material

# Supplementary Data

# Supplementary File 1. Nucleotide sequence alignment of MHCII *DRA* exon 2 in 70 Mongolian Bactrian camels.

>T1

AGGAACACGTGATCATCCAGGCTGAGTTCTATCTGAACCCTGACAAGTCA

GGCGAGTTTATGTTTGACTTTGACGGTGATGAGATTTTCCACGTGGATCT

GGAAAAGAAGGAGACGGTCTGGCGGCTTGAAGAATTTGGACGGTTTGCCA

GCTTTGAGGCTCAGGGTGCATTGGCCAATATAGCTGTGGACAAAGCCAAC

CTGGACATCATGATAAAGCGCTCCAACCACACCCCGAACACCAATG

>T2

AGGAACACGTGATCATCCAGGCTGAGTTCTATCTGAACCCTGACAAGTCA

GGCGAGTWTATGTTTGACTTTGACGGTGATGAGATTTTCCACGTGGATCT

GGAAAAGAAGGAGACGGTCTGGCGGCTTGAAGAATTTGGACGKTTTGCCA

GCTTTGAGGCTCAGGGTGCATTGGCCAATATAGCTGTGGACAAAGCCAAC

CTGGACATCATGATAAAGCGCTCCAACCACACCCCGAACACCAATG

>T3

AGGAACACGTGATCATCCAGGCTGAGTTCTATCTGAACCCTGACAAGTCA

GGCGAGTTTATGTTTGACTTTGACGGTGATGAGATTTTCCACGTGGATCT

GGAAAAGAAGGAGACGGTCTGGCGGCTTGAAGAATTTGGACGTTTTGCCA

GCTTTGAGGCTCAGGGTGCATTGGCCAATATAGCTGTGGACAAAGCCAAC

CTGGACATCATGATAAAGCGCTCCAACCACACCCCGAACACCAATG

>T5

AGGAACACGTGATCATCCAGGCTGAGTTCTATCTGAACCCTGACAAGTCA

GGCGAGTTTATGTTTGACTTTGACGGTGATGAGATTTTCCACGTGGATCT

GGAAAAGAAGGAGACGGTCTGGCGGCTTGAAGAATTTGGACGGTTTGCCA

GCTTTGAGGCTCAGGGTGCATTGGCCAATATAGCTGTGGACAAAGCCAAC

CTGGACATCATGATAAAGCGCTCCAACCACACCCCGAACACCAATG

>T6

AGGAACACGTGATCATCCAGGCTGAGTTCTATCTGAACCCTGACAAGTCA

GGCGAGTTTATGTTTGACTTTGACGGTGATGAGATTTTCCACGTGGATCT

GGAAAAGAAGGAGACGGTCTGGCGGCTTGAAGAATTTGGACGGTTTGCCA

GCTTTGAGGCTCAGGGTGCATTGGCCAATATAGCTGTGGACAAAGCCAAC

CTGGACATCATGATAAAGCGCTCCAACCACACCCCGAACACCAATG

>T7

AGGAACACGTGATCATCCAGGCTGAGTTCTATCTGAACCCTGACAAGTCA

GGCGAGTATATGTTTGACTTTGACGGTGATGAGATTTTCCACGTGGATCT

GGAAAAGAAGGAGACGGTCTGGCGGCTTGAAGAATTTGGACGTTTTGCCA

GCTTTGAGGCTCAGGGTGCATTGGCCAATATAGCTGTGGACAAAGCCAAC

CTGGACATCATGATAAAGCGCTCCAACCACACCCCGAACACCAATG

>T8

AGGAACACGTGATCATCCAGGCTGAGTTCTATCTGAACCCTGACAAGTCA

GGCGAGTATATGTTTGACTTTGACGGTGATGAGATTTTCCACGTGGATCT

GGAAAAGAAGGAGACGGTCTGGCGGCTTGAAGAATTTGGACGTTTTGCCA

GCTTTGAGGCTCAGGGTGCATTGGCCAATATAGCTGTGGACAAAGCCAAC

CTGGACATCATGATAAAGCGCTCCAACCACACCCCGAACACCAATG

>T9

AGGAACACGTGATCATCCAGGCTGAGTTCTATCTGAACCCTGACAAGTCA

GGCGAGTTTATGTTTGACTTTGACGGTGATGAGATTTTCCACGTGGATCT

GGAAAAGAAGGAGACGGTCTGGCGGCTTGAAGAATTTGGACGGTTTGCCA

GCTTTGAGGCTCAGGGTGCATTGGCCAATATAGCTGTGGACAAAGCCAAC

CTGGACATCATGATAAAGCGCTCCAACCACACCCCGAACACCAATG

>T10

AGGAACACGTGATCATCCAGGCTGAGTTCTATCTGAACCCTGACAAGTCA

GGCGAGTwTATGTTTGACTTTGACGGTGATGAGATTTTCCACGTGGATCT

GGAAAAGAAGGAGACGGTCTGGCGGCTTGAAGAATTTGGACGTTTTGCCA

GCTTTGAGGCTCAGGGTGCATTGGCCAATATAGCTGTGGACAAAGCCAAC

CTGGACATCATGATAAAGCGCTCCAACCACACCCCGAACACCAATG

>T11

AGGAACACGTGATCATCCAGGCTGAGTTCTATCTGAACCCTGACAAGTCA

GGCGAGTTTATGTTTGACTTTGACGGTGATGAGATTTTCCACGTGGATCT

GGAAAAGAAGGAGACGGTCTGGCGGCTTGAAGAATTTGGACGGTTTGCCA

GCTTTGAGGCTCAGGGTGCATTGGCCAATATAGCTGTGGACAAAGCCAAC

CTGGACATCATGATAAAGCGCTCCAACCACACCCCGAACACCAATG

>T12

AGGAACACGTGATCATCCAGGCTGAGTTCTATCTGAACCCTGACAAGTCA

GGCGAGTTTATGTTTGACTTTGACGGTGATGAGATTTTCCACGTGGATCT

GGAAAAGAAGGAGACGGTCTGGCGGCTTGAAGAATTTGGACGGTTTGCCA

GCTTTGAGGCTCAGGGTGCATTGGCCAATATAGCTGTGGACAAAGCCAAC

CTGGACATCATGATAAAGCGCTCCAACCACACCCCGAACACCAATG

>T13

AGGAACACGTGATCATCCAGGCTGAGTTCTATCTGAACCCTGACAAGTCA

GGCGAGTTTATGTTTGACTTTGACGGTGATGAGATTTTCCACGTGGATCT

GGAAAAGAAGGAGACGGTCTGGCGGCTTGAAGAATTTGGACGGTTTGCCA

GCTTTGAGGCTCAGGGTGCATTGGCCAATATAGCTGTGGACAAAGCCAAC

CTGGACATCATGATAAAGCGCTCCAACCACACCCCGAACACCAATG

>T14

AGGAACACGTGATCATCCAGGCTGAGTTCTATCTGAACCCTGACAAGTCA

GGCGAGTTTATGTTTGACTTTGACGGTGATGAGATTTTCCACGTGGATCT

GGAAAAGAAGGAGACGGTCTGGCGGCTTGAAGAATTTGGACGGTTTGCCA

GCTTTGAGGCTCAGGGTGCATTGGCCAATATAGCTGTGGACAAAGCCAAC

CTGGACATCATGATAAAGCGCTCCAACCACACCCCGAACACCAATG

>T15

AGGAACACGTGATCATCCAGGCTGAGTTCTATCTGAACCCTGACAAGTCA

GGCGAGTTTATGTTTGACTTTGACGGTGATGAGATTTTCCACGTGGATCT

GGAAAAGAAGGAGACGGTCTGGCGGCTTGAAGAATTTGGACGGTTTGCCA

GCTTTGAGGCTCAGGGTGCATTGGCCAATATAGCTGTGGACAAAGCCAAC

CTGGACATCATGATAAAGCGCTCCAACCACACCCCGAACACCAATG

>T23

AGGAACACGTGATCATCCAGGCTGAGTTCTATCTGAACCCTGACAAGTCA

GGCGAGTWTATGTTTGACTTTGACGGTGATGAGATTTTCCACGTGGATCT

GGAAAAGAAGGAGACGGTCTGGCGGCTTGAAGAATTTGGACGTTTTGCCA

GCTTTGAGGCTCAGGGTGCATTGGCCAATATAGCTGTGGACAAAGCCAAC

CTGGACATCATGATAAAGCGCTCCAACCACACCCCGAACACCAATG

>T24

AGGAACACGTGATCATCCAGGCTGAGTTCTATCTGAACCCTGACAAGTCA

GGCGAGTWTATGTTTGACTTTGACGGTGATGAGATTTTCCACGTGGATCT

GGAAAAGAAGGAGACGGTCTGGCGGCTTGAAGAATTTGGACGKTTTGCCA

GCTTTGAGGCTCAGGGTGCATTGGCCAATATAGCTGTGGACAAAGCCAAC

CTGGACATCATGATAAAGCGCTCCAACCACACCCCGAACACCAATG

>T25

AGGAACACGTGATCATCCAGGCTGAGTTCTATCTGAACCCTGACAAGTCA

GGCGAGTWTATGTTTGACTTTGACGGTGATGAGATTTTCCACGTGGATCT

GGAAAAGAAGGAGACGGTCTGGCGGCTTGAAGAATTTGGACGKTTTGCCA

GCTTTGAGGCTCAGGGTGCATTGGCCAATATAGCTGTGGACAAAGCCAAC

CTGGACATCATGATAAAGCGCTCCAACCACACCCCGAACACCAATG

>T26

AGGAACACGTGATCATCCAGGCTGAGTTCTATCTGAACCCTGACAAGTCA

GGCGAGTWTATGTTTGACTTTGACGGTGATGAGATTTTCCACGTGGATCT

GGAAAAGAAGGAGACGGTCTGGCGGCTTGAAGAATTTGGACGKTTTGCCA

GCTTTGAGGCTCAGGGTGCATTGGCCAATATAGCTGTGGACAAAGCCAAC

CTGGACATCATGATAAAGCGCTCCAACCACACCCCGAACACCAATG

>T27

AGGAACACGTGATCATCCAGGCTGAGTTCTATCTGAACCCTGACAAGTCA

GGCGAGTwTATGTTTGACTTTGACGGTGATGAGATTTTCCACGTGGATCT

GGAAAAGAAGGAGACGGTCTGGCGGCTTGAAGAATTTGGACGkTTTGCCA

GCTTTGAGGCTCAGGGTGCATTGGCCAATATAGCTGTGGACAAAGCCAAC

CTGGACATCATGATAAAGCGCTCCAACCACACCCCGAACACCAATG

>T28

AGGAACACGTGATCATCCAGGCTGAGTTCTATCTGAACCCTGACAAGTCA

GGCGAGTWTATGTTTGACTTTGACGGTGATGAGATTTTCCACGTGGATCT

GGAAAAGAAGGAGACGGTCTGGCGGCTTGAAGAATTTGGACGKTTTGCCA

GCTTTGAGGCTCAGGGTGCATTGGCCAATATAGCTGTGGACAAAGCCAAC

CTGGACATCATGATAAAGCGCTCCAACCACACCCCGAACACCAATG

>T29

AGGAACACGTGATCATCCAGGCTGAGTTCTATCTGAACCCTGACAAGTCA

GGCGAGTWTATGTTTGACTTTGACGGTGATGAGATTTTCCACGTGGATCT

GGAAAAGAAGGAGACGGTCTGGCGGCTTGAAGAATTTGGACGKTTTGCCA

GCTTTGAGGCTCAGGGTGCATTGGCCAATATAGCTGTGGACAAAGCCAAC

CTGGACATCATGATAAAGCGCTCCAACCACACCCCGAACACCAATG

>T30

AGGAACACGTGATCATCCAGGCTGAGTTCTATCTGAACCCTGACAAGTCA

GGCGAGTWTATGTTTGACTTTGACGGTGATGAGATTTTCCACGTGGATCT

GGAAAAGAAGGAGACGGTCTGGCGGCTTGAAGAATTTGGACGKTTTGCCA

GCTTTGAGGCTCAGGGTGCATTGGCCAATATAGCTGTGGACAAAGCCAAC

CTGGACATCATGATAAAGCGCTCCAACCACACCCCGAACACCAATG

>T31

AGGAACACGTGATCATCCAGGCTGAGTTCTATCTGAACCCTGACAAGTCA

GGCGAGTATATGTTTGACTTTGACGGTGATGAGATTTTCCACGTGGATCT

GGAAAAGAAGGAGACGGTCTGGCGGCTTGAAGAATTTGGACGTTTTGCCA

GCTTTGAGGCTCAGGGTGCATTGGCCAATATAGCTGTGGACAAAGCCAAC

CTGGACATCATGATAAAGCGCTCCAACCACACCCCGAACACCAATG

>T32

AGGAACACGTGATCATCCAGGCTGAGTTCTATCTGAACCCTGACAAGTCA

GGCGAGTWTATGTTTGACTTTGACGGTGATGAGATTTTCCACGTGGATCT

GGAAAAGAAGGAGACGGTCTGGCGGCTTGAAGAATTTGGACGKTTTGCCA

GCTTTGAGGCTCAGGGTGCATTGGCCAATATAGCTGTGGACAAAGCCAAC

CTGGACATCATGATAAAGCGCTCCAACCACACCCCGAACACCAATG

>T33

AGGAACACGTGATCATCCAGGCTGAGTTCTATCTGAACCCTGACAAGTCA

GGCGAGTWTATGTTTGACTTTGACGGTGATGAGATTTTCCACGTGGATCT

GGAAAAGAAGGAGACGGTCTGGCGGCTTGAAGAATTTGGACGKTTTGCCA

GCTTTGAGGCTCAGGGTGCATTGGCCAATATAGCTGTGGACAAAGCCAAC

CTGGACATCATGATAAAGCGCTCCAACCACACCCCGAACACCAATG

>T34

AGGAACACGTGATCATCCAGGCTGAGTTCTATCTGAACCCTGACAAGTCA

GGCGAGTWTATGTTTGACTTTGACGGTGATGAGATTTTCCACGTGGATCT

GGAAAAGAAGGAGACGGTCTGGCGGCTTGAAGAATTTGGACGKTTTGCCA

GCTTTGAGGCTCAGGGTGCATTGGCCAATATAGCTGTGGACAAAGCCAAC

CTGGACATCATGATAAAGCGCTCCAACCACACCCCGAACACCAATG

>T35

AGGAACACGTGATCATCCAGGCTGAGTTCTATCTGAACCCTGACAAGTCA

GGCGAGTATATGTTTGACTTTGACGGTGATGAGATTTTCCACGTGGATCT

GGAAAAGAAGGAGACGGTCTGGCGGCTTGAAGAATTTGGACGTTTTGCCA

GCTTTGAGGCTCAGGGTGCATTGGCCAATATAGCTGTGGACAAAGCCAAC

CTGGACATCATGATAAAGCGCTCCAACCACACCCCGAACACCAATG

>T36

AGGAACACGTGATCATCCAGGCTGAGTTCTATCTGAACCCTGACAAGTCA

GGCGAGTATATGTTTGACTTTGACGGTGATGAGATTTTCCACGTGGATCT

GGAAAAGAAGGAGACGGTCTGGCGGCTTGAAGAATTTGGACGTTTTGCCA

GCTTTGAGGCTCAGGGTGCATTGGCCAATATAGCTGTGGACAAAGCCAAC

CTGGACATCATGATAAAGCGCTCCAACCACACCCCGAACACCAATG

>T37

AGGAACACGTGATCATCCAGGCTGAGTTCTATCTGAACCCTGACAAGTCA

GGCGAGTTTATGTTTGACTTTGACGGTGATGAGATTTTCCACGTGGATCT

GGAAAAGAAGGAGACGGTCTGGCGGCTTGAAGAATTTGGACGGTTTGCCA

GCTTTGAGGCTCAGGGTGCATTGGCCAATATAGCTGTGGACAAAGCCAAC

CTGGACATCATGATAAAGCGCTCCAACCACACCCCGAACACCAATG

>T38

AGGAACACGTGATCATCCAGGCTGAGTTCTATCTGAACCCTGACAAGTCA

GGCGAGTWTATGTTTGACTTTGACGGTGATGAGATTTTCCACGTGGATCT

GGAAAAGAAGGAGACGGTCTGGCGGCTTGAAGAATTTGGACGKTTTGCCA

GCTTTGAGGCTCAGGGTGCATTGGCCAATATAGCTGTGGACAAAGCCAAC

CTGGACATCATGATAAAGCGCTCCAACCACACCCCGAACACCAATG

>T46

AGGAACACGTGATCATCCAGGCTGAGTTCTATCTGAACCCTGACAAGTCA

GGCGAGTATATGTTTGACTTTGACGGTGATGAGATTTTCCACGTGGATCT

GGAAAAGAAGGAGACGGTCTGGCGGCTTGAAGAATTTGGACGTTTTGCCA

GCTTTGAGGCTCAGGGTGCATTGGCCAATATAGCTGTGGACAAAGCCAAC

CTGGACATCATGATAAAGCGCTCCAACCACACCCCGAACACCAATG

>T54

AGGAACACGTGATCATCCAGGCTGAGTTCTATCTGAACCCTGACAAGTCA

GGCGAGTTTATGTTTGACTTTGACGGTGATGAGATTTTCCACGTGGATCT

GGAAAAGAAGGAGACGGTCTGGCGGCTTGAAGAATTTGGACGGTTTGCCA

GCTTTGAGGCTCAGGGTGCATTGGCCAATATAGCTGTGGACAAAGCCAAC

CTGGACATCATGATAAAGCGCTCCAACCACACCCCGAACACCAATG

>T55

AGGAACACGTGATCATCCAGGCTGAGTTCTATCTGAACCCTGACAAGTCA

GGCGAGTWTATGTTTGACTTTGACGGTGATGAGATTTTCCACGTGGATCT

GGAAAAGAAGGAGACGGTCTGGCGGCTTGAAGAATTTGGACGKTTTGCCA

GCTTTGAGGCTCAGGGTGCATTGGCCAATATAGCTGTGGACAAAGCCAAC

CTGGACATCATGATAAAGCGCTCCAACCACACCCCGAACACCAATG

>T56

AGGAACACGTGATCATCCAGGCTGAGTTCTATCTGAACCCTGACAAGTCA

GGCGAGTWTATGTTTGACTTTGACGGTGATGAGATTTTCCACGTGGATCT

GGAAAAGAAGGAGACGGTCTGGCGGCTTGAAGAATTTGGACGTTTTGCCA

GCTTTGAGGCTCAGGGTGCATTGGCCAATATAGCTGTGGACAAAGCCAAC

CTGGACATCATGATAAAGCGCTCCAACCACACCCCGAACACCAATG

>T57

AGGAACACGTGATCATCCAGGCTGAGTTCTATCTGAACCCTGACAAGTCA

GGCGAGTWTATGTTTGACTTTGACGGTGATGAGATTTTCCACGTGGATCT

GGAAAAGAAGGAGACGGTCTGGCGGCTTGAAGAATTTGGACGKTTTGCCA

GCTTTGAGGCTCAGGGTGCATTGGCCAATATAGCTGTGGACAAAGCCAAC

CTGGACATCATGATAAAGCGCTCCAACCACACCCCGAACACCAATG

>T58

AGGAACACGTGATCATCCAGGCTGAGTTCTATCTGAACCCTGACAAGTCA

GGCGAGTTTATGTTTGACTTTGACGGTGATGAGATTTTCCACGTGGATCT

GGAAAAGAAGGAGACGGTCTGGCGGCTTGAAGAATTTGGACGGTTTGCCA

GCTTTGAGGCTCAGGGTGCATTGGCCAATATAGCTGTGGACAAAGCCAAC

CTGGACATCATGATAAAGCGCTCCAACCACACCCCGAACACCAATG

>T59

AGGAACACGTGATCATCCAGGCTGAGTTCTATCTGAACCCTGACAAGTCA

GGCGAGTATATGTTTGACTTTGACGGTGATGAGATTTTCCACGTGGATCT

GGAAAAGAAGGAGACGGTCTGGCGGCTTGAAGAATTTGGACGTTTTGCCA

GCTTTGAGGCTCAGGGTGCATTGGCCAATATAGCTGTGGACAAAGCCAAC

CTGGACATCATGATAAAGCGCTCCAACCACACCCCGAACACCAATG

>T60

AGGAACACGTGATCATCCAGGCTGAGTTCTATCTGAACCCTGACAAGTCA

GGCGAGTWTATGTTTGACTTTGACGGTGATGAGATTTTCCACGTGGATCT

GGAAAAGAAGGAGACGGTCTGGCGGCTTGAAGAATTTGGACGTTTTGCCA

GCTTTGAGGCTCAGGGTGCATTGGCCAATATAGCTGTGGACAAAGCCAAC

CTGGACATCATGATAAAGCGCTCCAACCACACCCCGAACACCAATG

>T61

AGGAACACGTGATCATCCAGGCTGAGTTCTATCTGAACCCTGACAAGTCA

GGCGAGTWTATGTTTGACTTTGACGGTGATGAGATTTTCCACGTGGATCT

GGAAAAGAAGGAGACGGTCTGGCGGCTTGAAGAATTTGGACGKTTTGCCA

GCTTTGAGGCTCAGGGTGCATTGGCCAATATAGCTGTGGACAAAGCCAAC

CTGGACATCATGATAAAGCGCTCCAACCACACCCCGAACACCAATG

>T62

AGGAACACGTGATCATCCAGGCTGAGTTCTATCTGAACCCTGACAAGTCA

GGCGAGTATATGTTTGACTTTGACGGTGATGAGATTTTCCACGTGGATCT

GGAAAAGAAGGAGACGGTCTGGCGGCTTGAAGAATTTGGACGTTTTGCCA

GCTTTGAGGCTCAGGGTGCATTGGCCAATATAGCTGTGGACAAAGCCAAC

CTGGACATCATGATAAAGCGCTCCAACCACACCCCGAACACCAATG

>T63

AGGAACACGTGATCATCCAGGCTGAGTTCTATCTGAACCCTGACAAGTCA

GGCGAGTWTATGTTTGACTTTGACGGTGATGAGATTTTCCACGTGGATCT

GGAAAAGAAGGAGACGGTCTGGCGGCTTGAAGAATTTGGACGKTTTGCCA

GCTTTGAGGCTCAGGGTGCATTGGCCAATATAGCTGTGGACAAAGCCAAC

CTGGACATCATGATAAAGCGCTCCAACCACACCCCGAACACCAATG

>T64

AGGAACACGTGATCATCCAGGCTGAGTTCTATCTGAACCCTGACAAGTCA

GGCGAGTWTATGTTTGACTTTGACGGTGATGAGATTTTCCACGTGGATCT

GGAAAAGAAGGAGACGGTCTGGCGGCTTGAAGAATTTGGACGKTTTGCCA

GCTTTGAGGCTCAGGGTGCATTGGCCAATATAGCTGTGGACAAAGCCAAC

CTGGACATCATGATAAAGCGCTCCAACCACACCCCGAACACCAATG

>T65

AGGAACACGTGATCATCCAGGCTGAGTTCTATCTGAACCCTGACAAGTCA

GGCGAGTTTATGTTTGACTTTGACGGTGATGAGATTTTCCACGTGGATCT

GGAAAAGAAGGAGACGGTCTGGCGGCTTGAAGAATTTGGACGKTTTGCCA

GCTTTGAGGCTCAGGGTGCATTGGCCAATATAGCTGTGGACAAAGCCAAC

CTGGACATCATGATAAAGCGCTCCAACCACACCCCGAACACCAATG

>T66

AGGAACACGTGATCATCCAGGCTGAGTTCTATCTGAACCCTGACAAGTCA

GGCGAGTWTATGTTTGACTTTGACGGTGATGAGATTTTCCACGTGGATCT

GGAAAAGAAGGAGACGGTCTGGCGGCTTGAAGAATTTGGACGKTTTGCCA

GCTTTGAGGCTCAGGGTGCATTGGCCAATATAGCTGTGGACAAAGCCAAC

CTGGACATCATGATAAAGCGCTCCAACCACACCCCGAACACCAATG

>T67

AGGAACACGTGATCATCCAGGCTGAGTTCTATCTGAACCCTGACAAGTCA

GGCGAGTWTATGTTTGACTTTGACGGTGATGAGATTTTCCACGTGGATCT

GGAAAAGAAGGAGACGGTCTGGCGGCTTGAAGAATTTGGACGKTTTGCCA

GCTTTGAGGCTCAGGGTGCATTGGCCAATATAGCTGTGGACAAAGCCAAC

CTGGACATCATGATAAAGCGCTCCAACCACACCCCGAACACCAATG

>T68

AGGAACACGTGATCATCCAGGCTGAGTTCTATCTGAACCCTGACAAGTCA

GGCGAGTTTATGTTTGACTTTGACGGTGATGAGATTTTCCACGTGGATCT

GGAAAAGAAGGAGACGGTCTGGCGGCTTGAAGAATTTGGACGGTTTGCCA

GCTTTGAGGCTCAGGGTGCATTGGCCAATATAGCTGTGGACAAAGCCAAC

CTGGACATCATGATAAAGCGCTCCAACCACACCCCGAACACCAATG

>T69

AGGAACACGTGATCATCCAGGCTGAGTTCTATCTGAACCCTGACAAGTCA

GGCGAGTWTATGTTTGACTTTGACGGTGATGAGATTTTCCACGTGGATCT

GGAAAAGAAGGAGACGGTCTGGCGGCTTGAAGAATTTGGACGTTTTGCCA

GCTTTGAGGCTCAGGGTGCATTGGCCAATATAGCTGTGGACAAAGCCAAC

CTGGACATCATGATAAAGCGCTCCAACCACACCCCGAACACCAATG

>T70

AGGAACACGTGATCATCCAGGCTGAGTTCTATCTGAACCCTGACAAGTCA

GGCGAGTTTATGTTTGACTTTGACGGTGATGAGATTTTCCACGTGGATCT

GGAAAAGAAGGAGACGGTCTGGCGGCTTGAAGAATTTGGACGKTTTGCCA

GCTTTGAGGCTCAGGGTGCATTGGCCAATATAGCTGTGGACAAAGCCAAC

CTGGACATCATGATAAAGCGCTCCAACCACACCCCGAACACCAATG

>T71

AGGAACACGTGATCATCCAGGCTGAGTTCTATCTGAACCCTGACAAGTCA

GGCGAGTWTATGTTTGACTTTGACGGTGATGAGATTTTCCACGTGGATCT

GGAAAAGAAGGAGACGGTCTGGCGGCTTGAAGAATTTGGACGKTTTGCCA

GCTTTGAGGCTCAGGGTGCATTGGCCAATATAGCTGTGGACAAAGCCAAC

CTGGACATCATGATAAAGCGCTCCAACCACACCCCGAACACCAATG

>T72

AGGAACACGTGATCATCCAGGCTGAGTTCTATCTGAACCCTGACAAGTCA

GGCGAGTWTATGTTTGACTTTGACGGTGATGAGATTTTCCACGTGGATCT

GGAAAAGAAGGAGACGGTCTGGCGGCTTGAAGAATTTGGACGTTTTGCCA

GCTTTGAGGCTCAGGGTGCATTGGCCAATATAGCTGTGGACAAAGCCAAC

CTGGACATCATGATAAAGCGCTCCAACCACACCCCGAACACCAATG

>T73

AGGAACACGTGATCATCCAGGCTGAGTTCTATCTGAACCCTGACAAGTCA

GGCGAGTWTATGTTTGACTTTGACGGTGATGAGATTTTCCACGTGGATCT

GGAAAAGAAGGAGACGGTCTGGCGGCTTGAAGAATTTGGACGKTTTGCCA

GCTTTGAGGCTCAGGGTGCATTGGCCAATATAGCTGTGGACAAAGCCAAC

CTGGACATCATGATAAAGCGCTCCAACCACACCCCGAACACCAATG

>T74

AGGAACACGTGATCATCCAGGCTGAGTTCTATCTGAACCCTGACAAGTCA

GGCGAGTWTATGTTTGACTTTGACGGTGATGAGATTTTCCACGTGGATCT

GGAAAAGAAGGAGACGGTCTGGCGGCTTGAAGAATTTGGACGTTTTGCCA

GCTTTGAGGCTCAGGGTGCATTGGCCAATATAGCTGTGGACAAAGCCAAC

CTGGACATCATGATAAAGCGCTCCAACCACACCCCGAACACCAATG

>T76

AGGAACACGTGATCATCCAGGCTGAGTTCTATCTGAACCCTGACAAGTCA

GGCGAGTATATGTTTGACTTTGACGGTGATGAGATTTTCCACGTGGATCT

GGAAAAGAAGGAGACGGTCTGGCGGCTTGAAGAATTTGGACGTTTTGCCA

GCTTTGAGGCTCAGGGTGCATTGGCCAATATAGCTGTGGACAAAGCCAAC

CTGGACATCATGATAAAGCGCTCCAACCACACCCCGAACACCAATG

>T77

AGGAACACGTGATCATCCAGGCTGAGTTCTATCTGAACCCTGACAAGTCA

GGCGAGTATATGTTTGACTTTGACGGTGATGAGATTTTCCACGTGGATCT

GGAAAAGAAGGAGACGGTCTGGCGGCTTGAAGAATTTGGACGTTTTGCCA

GCTTTGAGGCTCAGGGTGCATTGGCCAATATAGCTGTGGACAAAGCCAAC

CTGGACATCATGATAAAGCGCTCCAACCACACCCCGAACACCAATG

>T78

AGGAACACGTGATCATCCAGGCTGAGTTCTATCTGAACCCTGACAAGTCA

GGCGAGTATATGTTTGACTTTGACGGTGATGAGATTTTCCACGTGGATCT

GGAAAAGAAGGAGACGGTCTGGCGGCTTGAAGAATTTGGACGTTTTGCCA

GCTTTGAGGCTCAGGGTGCATTGGCCAATATAGCTGTGGACAAAGCCAAC

CTGGACATCATGATAAAGCGCTCCAACCACACCCCGAACACCAATG

>T79

AGGAACACGTGATCATCCAGGCTGAGTTCTATCTGAACCCTGACAAGTCA

GGCGAGTWTATGTTTGACTTTGACGGTGATGAGATTTTCCACGTGGATCT

GGAAAAGAAGGAGACGGTCTGGCGGCTTGAAGAATTTGGACGTTTTGCCA

GCTTTGAGGCTCAGGGTGCATTGGCCAATATAGCTGTGGACAAAGCCAAC

CTGGACATCATGATAAAGCGCTCCAACCACACCCCGAACACCAATG

>T80

AGGAACACGTGATCATCCAGGCTGAGTTCTATCTGAACCCTGACAAGTCA

GGCGAGTWTATGTTTGACTTTGACGGTGATGAGATTTTCCACGTGGATCT

GGAAAAGAAGGAGACGGTCTGGCGGCTTGAAGAATTTGGACGKTTTGCCA

GCTTTGAGGCTCAGGGTGCATTGGCCAATATAGCTGTGGACAAAGCCAAC

CTGGACATCATGATAAAGCGCTCCAACCACACCCCGAACACCAATG

>T81

AGGAACACGTGATCATCCAGGCTGAGTTCTATCTGAACCCTGACAAGTCA

GGCGAGTWTATGTTTGACTTTGACGGTGATGAGATTTTCCACGTGGATCT

GGAAAAGAAGGAGACGGTCTGGCGGCTTGAAGAATTTGGACGTTTTGCCA

GCTTTGAGGCTCAGGGTGCATTGGCCAATATAGCTGTGGACAAAGCCAAC

CTGGACATCATGATAAAGCGCTCCAACCACACCCCGAACACCAATG

>T82

AGGAACACGTGATCATCCAGGCTGAGTTCTATCTGAACCCTGACAAGTCA

GGCGAGTWTATGTTTGACTTTGACGGTGATGAGATTTTCCACGTGGATCT

GGAAAAGAAGGAGACGGTCTGGCGGCTTGAAGAATTTGGACGTTTTGCCA

GCTTTGAGGCTCAGGGTGCATTGGCCAATATAGCTGTGGACAAAGCCAAC

CTGGACATCATGATAAAGCGCTCCAACCACACCCCGAACACCAATG

>T84

AGGAACACGTGATCATCCAGGCTGAGTTCTATCTGAACCCTGACAAGTCA

GGCGAGTWTATGTTTGACTTTGACGGTGATGAGATTTTCCACGTGGATCT

GGAAAAGAAGGAGACGGTCTGGCGGCTTGAAGAATTTGGACGKTTTGCCA

GCTTTGAGGCTCAGGGTGCATTGGCCAATATAGCTGTGGACAAAGCCAAC

CTGGACATCATGATAAAGCGCTCCAACCACACCCCGAACACCAATG

>T85

AGGAACACGTGATCATCCAGGCTGAGTTCTATCTGAACCCTGACAAGTCA

GGCGAGTWTATGTTTGACTTTGACGGTGATGAGATTTTCCACGTGGATCT

GGAAAAGAAGGAGACGGTCTGGCGGCTTGAAGAATTTGGACGKTTTGCCA

GCTTTGAGGCTCAGGGTGCATTGGCCAATATAGCTGTGGACAAAGCCAAC

CTGGACATCATGATAAAGCGCTCCAACCACACCCCGAACACCAATG

>T86

AGGAACACGTGATCATCCAGGCTGAGTTCTATCTGAACCCTGACAAGTCA

GGCGAGTATATGTTTGACTTTGACGGTGATGAGATTTTCCACGTGGATCT

GGAAAAGAAGGAGACGGTCTGGCGGCTTGAAGAATTTGGACGTTTTGCCA

GCTTTGAGGCTCAGGGTGCATTGGCCAATATAGCTGTGGACAAAGCCAAC

CTGGACATCATGATAAAGCGCTCCAACCACACCCCGAACACCAATG

>T87

AGGAACACGTGATCATCCAGGCTGAGTTCTATCTGAACCCTGACAAGTCA

GGCGAGTWTATGTTTGACTTTGACGGTGATGAGATTTTCCACGTGGATCT

GGAAAAGAAGGAGACGGTCTGGCGGCTTGAAGAATTTGGACGKTTTGCCA

GCTTTGAGGCTCAGGGTGCATTGGCCAATATAGCTGTGGACAAAGCCAAC

CTGGACATCATGATAAAGCGCTCCAACCACACCCCGAACACCAATG

>T88

AGGAACACGTGATCATCCAGGCTGAGTTCTATCTGAACCCTGACAAGTCA

GGCGAGTWTATGTTTGACTTTGACGGTGATGAGATTTTCCACGTGGATCT

GGAAAAGAAGGAGACGGTCTGGCGGCTTGAAGAATTTGGACGTTTTGCCA

GCTTTGAGGCTCAGGGTGCATTGGCCAATATAGCTGTGGACAAAGCCAAC

CTGGACATCATGATAAAGCGCTCCAACCACACCCCGAACACCAATG

>T89

AGGAACACGTGATCATCCAGGCTGAGTTCTATCTGAACCCTGACAAGTCA

GGCGAGTTTATGTTTGACTTTGACGGTGATGAGATTTTCCACGTGGATCT

GGAAAAGAAGGAGACGGTCTGGCGGCTTGAAGAATTTGGACGTTTTGCCA

GCTTTGAGGCTCAGGGTGCATTGGCCAATATAGCTGTGGACAAAGCCAAC

CTGGACATCATGATAAAGCGCTCCAACCACACCCCGAACACCAATG

>T90

AGGAACACGTGATCATCCAGGCTGAGTTCTATCTGAACCCTGACAAGTCA

GGCGAGTWTATGTTTGACTTTGACGGTGATGAGATTTTCCACGTGGATCT

GGAAAAGAAGGAGACGGTCTGGCGGCTTGAAGAATTTGGACGKTTTGCCA

GCTTTGAGGCTCAGGGTGCATTGGCCAATATAGCTGTGGACAAAGCCAAC

CTGGACATCATGATAAAGCGCTCCAACCACACCCCGAACACCAATG

>T177

AGGAACACGTGATCATCCAGGCTGAGTTCTATCTGAACCCTGACAAGTCA

GGCGAGTWTATGTTTGACTTTGACGGTGATGAGATTTTCCACGTGGATCT

GGAAAAGAAGGAGACGGTCTGGCGGCTTGAAGAATTTGGACGTTTTGCCA

GCTTTGAGGCTCAGGGTGCATTGGCCAATATAGCTGTGGACAAAGCCAAC

CTGGACATCATGATAAAGCGCTCCAACCACACCCCGAACACCAATG

>T178

AGGAACACGTGATCATCCAGGCTGAGTTCTATCTGAACCCTGACAAGTCA

GGCGAGTTTATGTTTGACTTTGACGGTGATGAGATTTTCCACGTGGATCT

GGAAAAGAAGGAGACGGTCTGGCGGCTTGAAGAATTTGGACGKTTTGCCA

GCTTTGAGGCTCAGGGTGCATTGGCCAATATAGCTGTGGACAAAGCCAAC

CTGGACATCATGATAAAGCGCTCCAACCACACCCCGAACACCAATG

>T179

AGGAACACGTGATCATCCAGGCTGAGTTCTATCTGAACCCTGACAAGTCA

GGCGAGTTTATGTTTGACTTTGACGGTGATGAGATTTTCCACGTGGATCT

GGAAAAGAAGGAGACGGTCTGGCGGCTTGAAGAATTTGGACGKTTTGCCA

GCTTTGAGGCTCAGGGTGCATTGGCCAATATAGCTGTGGACAAAGCCAAC

CTGGACATCATGATAAAGCGCTCCAACCACACCCCGAACACCAATG

>T180

AGGAACACGTGATCATCCAGGCTGAGTTCTATCTGAACCCTGACAAGTCA

GGCGAGTTTATGTTTGACTTTGACGGTGATGAGATTTTCCACGTGGATCT

GGAAAAGAAGGAGACGGTCTGGCGGCTTGAAGAATTTGGACGKTTTGCCA

GCTTTGAGGCTCAGGGTGCATTGGCCAATATAGCTGTGGACAAAGCCAAC

CTGGACATCATGATAAAGCGCTCCAACCACACCCCGAACACCAATG
